# Psychedelic‐ and Substance‐Assisted Therapies in Global Mental Health: Bridging Cultures, Evidence, and Access,

**DOI:** 10.1002/brb3.71265

**Published:** 2026-02-16

**Authors:** Simon Halm

**Affiliations:** ^1^ University of Zurich Zurich Switzerland

## Abstract

Psychedelic‐ and substance‐assisted therapies, including MDMA, psilocybin, and ketamine, are gaining attention for conditions such as PTSD and depression, yet their development and implementation remain largely concentrated in high‐income settings. This graphical abstract summarizes the central argument of the commentary: while these interventions may hold relevance for global mental health, particularly in conflict‐affected and humanitarian contexts, their equitable use is constrained by cultural, ethical, regulatory, and resource‐related challenges. Responsible implementation requires culturally grounded, ethically robust, and context‐sensitive pathways rather than uncritical expansion.

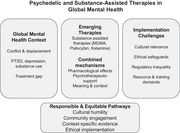

## 1 Introduction

Mental health is a global public good, affecting millions across all countries (Patel et al. 2018; Abbafati et al. 2020). Humanitarian emergencies, armed conflict, and displacement profoundly impact mental health and psychological well‐being (Turrini et al. 2017; Steel et al. 2009): The prevalence of disorders such as PTSD and depression can be up to 10 times higher in conflict or post‐conflict settings. Effective global mental healthcare requires that everyone, everywhere, has access to the care they need—even and especially during crises. However, despite advances in psychopharmacology and psychotherapy, many people experience only partial or no response to available treatments. This gap has driven renewed interest in substance‐assisted therapies as innovative interventions, particularly for treatment‐resistant depression and PTSD. Substances such as MDMA, psilocybin, and ketamine have demonstrated promising results in clinical trials, combining pharmacological and psychotherapeutic mechanisms. Yet, current research and clinical implementation remain largely confined to high‐income countries, raising questions about accessibility, cultural adaptation, and global relevance.

## 2 Historical and Cultural Roots of Psychedelic Use

The therapeutic use of psychoactive substances is not a new phenomenon. Long before their renaissance in Western psychiatry and neuroscience, psychedelic plants such as ayahuasca, iboga, and psilocybin‐containing mushrooms were used for spiritual and healing purposes by indigenous communities across the world. Many of these traditions emphasized communal, ritualized settings aimed at meaning‐making, social cohesion, and spiritual balance (George et al. 2022).

Modern research with psychedelics and other novel substances, largely driven by Western institutions, builds upon these traditions but medicalizes them within a biomedical framework. While focusing on symptom reduction and standardized protocols, it still acknowledges the importance of context and meaning. Nevertheless, some advocacy groups have even argued that Western medical use represents a form of cultural appropriation. Alternatively, a more constructive framing may be to view it as an opportunity to converge traditional and biomedical paradigms. The central challenge should not be ownership but ensuring equitable access, safety, and cultural appropriateness across diverse contexts.

## 3 Current Evidence and Regulatory Landscape

The evidence base for the clinical use of psychedelics and other novel substances has advanced rapidly over the past decade. MDMA, an entactogen rather than a classic psychedelic, is the most extensively studied compound for substance‐assisted psychotherapy for PTSD. In a recent Phase 3 trial, 71% of participants receiving MDMA‐assisted psychotherapy no longer met criteria for PTSD at study end, compared to 48% in the control group (Mitchell et al. 2021). However, concerns about bias, unblinding, and limited long‐term follow‐up led the US Food and Drug Administration (FDA) in 2024 to withhold approval, requesting further evidence (Kupferschmidt 2024).

Ketamine, which is approved globally as an anesthetic, is used off‐label in psychiatry and has shown transient symptom relief in PTSD (Borgogna et al. 2024). Its enantiomer, esketamine, has been approved in its intranasal formulation for treatment‐resistant depression in a number of high‐income jurisdictions, including the United States, Canada, the European Union, Switzerland, and Australia. Psilocybin, a serotonin 5‐HT_2A_ receptor agonist, demonstrates robust antidepressant effects (Raison et al. 2023; Carhart‐Harris et al. 2021; Rieser et al. 2025), though its use for PTSD remains in early stages. Other compounds such as LSD and DMT (often in ayahuasca form) are under exploration, but further randomized controlled data are needed.

Regulatory developments regarding clinical use vary widely. Switzerland allows MDMA‐, psilocybin‐, and LSD‐assisted therapy under strict, case‐by‐case special exemptions when there are no alternative treatments and has facilitated over a thousand individual treatments (Aicher et al. 2024). In Australia, MDMA can be prescribed exclusively for treatment‐resistant PTSD and psilocybin for treatment‐resistant depression, and Canada has similarly granted access for defined indications, such as psilocybin for major depressive disorder and end‐of‐life psychological distress, and MDMA for the treatment of PTSD. In contrast, most low‐ and middle‐income countries lack the legal and research infrastructure to explore these therapies in controlled settings. This uneven geography of innovation reinforces global health inequities, excluding populations most affected by conflict, poverty, and limited mental health infrastructure from innovative treatments.

## 4 Emerging Interest in Conflict‐Affected and Humanitarian Settings

At the same time, a number of middle‐income and crisis‐affected countries are beginning to engage with this evolving field. Growing interest in psychedelic‐assisted therapy in Ukraine illustrates how such contexts seek to participate in global developments despite limited resources and ongoing conflict. The Ukrainian Psychedelic Research Association (UPRA) has advocated for research and regulatory reform since 2022, supported by national and international partners. A pilot ketamine study for PTSD is underway, and proposals to reschedule MDMA and psilocybin for research are under review by the Ministry of Health (Hawrot 2025). This initiative reflects a wider global need to expand access to novel therapies for veterans and civilians affected by war‐related trauma.

Major humanitarian and UN organizations have integrated substantial mental health components into their programs from transdiagnostic interventions such as WHO's Problem Management Plus (PM+) to vertical projects targeting specific diagnoses such as PTSD. However, none are yet directly engaged in psychedelic‐assisted treatment. For these actors, Ukraine raises important questions about whether and how such therapies might fit within humanitarian mental health frameworks. Ketamine, already a WHO essential medicine, demonstrates that accessibility barriers are not only logistical or financial but also depend on safety protocols, evidence generation, and integration into culturally and ethically sound care models.

## 5 Challenges and Cultural Considerations

Several factors constrain equitable access to psychedelic‐assisted therapies. Most evidence comes from controlled research in the Global North with highly selected participants, limiting generalizability. Implementation requires specialized therapist training, controlled environments, and extensive integration sessions, all resource‐intensive components that are difficult to replicate in low‐resource or humanitarian contexts. These barriers are amplified by restrictive regulations, limited clinical infrastructure, and a scarcity of trained professionals. Other ethical uncertainties regarding informed consent, potential therapist misconduct, or exploitation might be amplified in low‐income settings and highlight the need for culturally appropriate and ethically robust models of care.

A deeper challenge concerns the *epistemological fit* of psychedelic‐ and substance‐assisted therapies with local conceptions of suffering, healing, and consciousness. Frameworks for understanding altered states of consciousness differ between societies, emphasizing the importance of local adaptations in research and clinical use. Without such cultural and conceptual alignment, interventions risk being perceived as foreign or incongruent, reducing both their acceptability and effectiveness.

Due to the spiritual and existential nature of some psychedelic experiences, religious aspects may play an important role in this regard. Religious and spiritual beliefs may provide interpretive frameworks that help make altered states of consciousness meaningful and psychologically integrated. In some contexts, engagement with religious worldviews or community leaders may therefore facilitate access, build trust, and support ethical implementation. Conversely, certain religious or moral frameworks may also constrain access by reinforcing moral prohibitions or stigma surrounding substance use.

## 6 Responsible Implementation

Ensuring the global appropriateness of psychedelic therapy is not only a matter of setting adaptation and resource distribution but also of underlying mindset. Healing frameworks differ widely across societies and introducing these substances without community engagement risks reproducing colonial or technocratic models of care. Conversely, denying access to innovative treatments on the grounds of cultural difference or socioeconomic status would reinforce inequities.

There is also potential for an inverse flow of knowledge: collective resilience, spirituality, and community‐based understandings of healing in low‐income settings may enhance the therapeutic integration of psychedelic experiences elsewhere. A balanced approach should aim for *cultural humility*—integrating local concepts of distress, consciousness, and recovery while maintaining adherence to evidence‐based safety standards.

Enthusiasm around psychedelics must also be informed by historical awareness. The premature popularization of LSD therapy in the 1960s provoked political backlash, setting research back for decades. Avoiding a repetition of that pattern requires careful implementation, transparent reporting, and open dialogue between scientific, regulatory, community, and humanitarian actors. If done cautiously, scalable adaptation seems possible. Compounds like ketamine could be integrated into existing systems where anesthesia and emergency care already exist, provided that evidence‐based mental health protocols, cultural adaptation, and safety measures accompany their use.

## 7 Way Forward

The renewed scientific interest in psychedelic‐ and substance‐assisted therapies offers a major opportunity for psychiatry and potentially for global mental health. Yet their benefits risk remaining confined to well‐resourced contexts unless deliberate efforts are made to expand research and access across diverse settings. Generating cross‐cultural and cross‐context evidence is essential not only to establish efficacy and safety but also to draw on contextual insights that may deepen understanding and enhance therapeutic effectiveness.

As the field progresses, collaboration between communities, academics, humanitarian organizations, and public health institutions should develop models of responsible and equitable implementation that align treatment development with global mental health priorities. Psychedelics may one day become important tools in the treatment of PTSD, depression, and substance use disorders, but their true potential will only be realized when those most affected by mental illness, often in low‐income, low‐resource, or crisis settings, are not the last to benefit.
